# Foundation for Bioproduction: Secretory Stages, Metabolite Profiles and Gene Function of Glandular Trichomes in Cucumber

**DOI:** 10.3390/ijms27073276

**Published:** 2026-04-04

**Authors:** Yuming Dong, Jiancai Mao, Xue Feng, Zhigang Tang, Li Shan, Sen Li, Yaru Wang, Yongdong Sun, Huazhong Ren, Xingwang Liu

**Affiliations:** 1 College of Horticulture, China Agricultural University, Beijing 100193, China; bs20233171036@cau.edu.cn (Y.D.); mjc_fly@foxmail.com (J.M.); fengxue917@163.com (X.F.); tzg2505787780@163.com (Z.T.); bs20233171035@cau.edu.cn (S.L.); 2 Institute of Fruits and Vegetables, Xinjiang Academy of Agricultural Sciences, Urumqi 832000, China; 3 Sanya Institute of China Agricultural University, Sanya 572000, China; shanli01@cau.edu.cn (L.S.); b20213170888@cau.edu.cn (Y.W.); 4 School of Horticulture and Landscape Architecture, Henan Institute of Science and Technology, Xinxiang 453003, China; sunyd2001@163.com

**Keywords:** cucumber, type I glandular trichomes, secretion process, ultrastructure, metabolite component

## Abstract

Glandular trichomes (GTs) are epidermal outgrowths that function as “natural cell factories” for the synthesis of specialized metabolites. Beyond their traditional understanding, GTs on cucumber fruits can form an undesirable trait known as bloom, which negatively affects market value. However, the secretory process, metabolite profiles, and genetic regulation underlying GT development in cucumber remain largely unclear. In this study, we employed scanning electron microscopy (SEM), transmission electron microscopy (TEM), histochemical staining, multi-omics analyses, and liquid chromatography–mass spectrometry (LC-MS) to systematically investigate GT development. The secretory process was classified into four distinct stages via SEM observations: morphogenesis, active metabolism, head sunken, and metabolite release. TEM revealed progressive ultrastructural changes, including increased organelle abundance and expansion of the periplasmic space, which facilitate metabolite transport and release. This process occurs through an autonomous mechanism involving osmiophilic substances and eventual cell rupture. LC-MS analysis identified 744 metabolites belonging to 11 classes, with phenylpropanoids/polyketides—particularly flavonoids—being the most abundant. While metabolite classes are conserved between European greenhouse and North China ecotypes, specific metabolite contents vary significantly. Multi-transcriptome analysis identified 60 candidate genes associated with GT development. Among these, *CsaV4_3G003418* was functionally validated through virus-induced gene silencing (VIGS) to be involved in early GT development. Collectively, this work elucidates the secretory mechanism and metabolic characteristics of cucumber GTs, providing a foundation for future functional studies and biotechnological applications of secondary metabolites.

## 1. Introduction

Cucumber (*Cucumis sativus* L.) is one of the most important horticultural crops widely cultivated globally, valued for its rich nutritional content and refreshing taste [1]. In China, both the cultivation area and the total yield of cucumber rank first in the world. The aerial surfaces of cucumber plants are covered with various types of trichomes, which are traditionally classified into glandular and non-glandular categories based on the presence or absence of secretory capacity [2,3]. The developmental processes of cucumber trichomes are divided into five stages [4]. Among these, glandular trichomes (GTs) are hypothesized to play a critical role in the synthesis and secretion of specialized metabolites, potentially contributing to pest resistance and environmental adaptation [5]. In addition, cucumber GTs are an important trait affecting the appearance of cucumber fruits. The previously published a key trait called “fruit bloom” is formed by the rupture of GTs [6]. Despite their morphological prominence and presumptive functional significance, the secretory mechanisms, ultrastructural dynamics, and metabolic profiles of GTs in cucumber remain poorly characterized.

In other plant species, GTs are specialized epidermal structures that synthesize a diverse range of secondary metabolites, including terpenoids [7], flavonoids [8], alkaloids, and acyl sugars [9], which often serve defensive, ecological, or commercial functions. In cultivated tomatoes (*Solanum lycopersicum*) and their wild relatives, Type I and Type IV glandular trichomes (GTs) are responsible for producing acyl sugars, which are secreted directly from the apex of glandular head cells. As the most abundant trichome type in tomato, Type VI trichomes mainly function in the biosynthesis of monoterpenoids [10]. Pyrethrins, produced by the GTs of pyrethrum (*Tanacetum cinerariifolium*), exhibit significant biological activity against a wide range of insects [11]. Carnosic acid, synthesized in the GTs of young leaves from plants such as rosemary and sage, protects chloroplasts from photooxidative stress [12]. Tobacco alkaloids (nicotine, nornicotine, anabasine, and other minor alkaloids) are biosynthesized in the roots and subsequently transported to aerial parts of tobacco plants, including GTs, where they function in defending against herbivorous insects, regulating plant growth, and detoxification [13]. Acyl sugars, produced and stored by GTs of various *Solanaceous* plants, act as excellent surfactants and emulsifiers that readily adhere to the cuticle of arthropods to trap insects and exhibit toxicity to herbivores [14,15].

In addition to their diverse biological functions, secondary metabolites derived from GTs have broad applications in the pharmaceutical, pesticide, and fragrance industries. GTs of *Lamiaceae* plants (e.g., basil, lavender, mint, oregano, and thyme) produce essential oils [16]. GTs of cannabis synthesize unique terpenophenolic compounds (cannabinoids): Δ^9^-tetrahydrocannabinol (THC) is a psychoactive cannabinoid with antiemetic and anticancer activities [17], while cannabidiol (CBD), a non-psychoactive cannabinoid, has been proven effective in preventing neurodegenerative and cardiovascular diseases [18]. Gossypol and related diterpenoids produced by cotton GTs exhibit antifungal activity and serve as potential natural insecticides [19]. GTs function as efficient “plant factories” for the production of high-value secondary metabolites, serving as promising targets for biotechnological exploitation and natural product development [20]. However, analogous investigations in cucumber are notably scarce, leaving a substantial knowledge gap regarding the biochemical and developmental basis of its GT functions. Elucidating the secretory process and metabolite composition of cucumber GTs are therefore essential not only for advancing fundamental understanding of GT biology but also for exploiting these structures in plant improvement and natural product discovery.

Herein, cucumber GTs were selected as the research object. Scanning electron microscopy (SEM) was used to observe morphology during their secretory process, which was divided into four distinct stages. Transmission electron microscopy (TEM) was employed to characterize the ultrastructural features of GTs at different secretory stages. Following the collection of a large quantity of GTs using bead beating, targeted metabolite profiling was conducted via liquid chromatography–mass spectrometry (LC-MS). Through virus-induced gene silencing (VIGS) assays, we confirmed that *CsaV4_3G003418* plays a crucial role in regulating the density of GTs. We hypothesized that (1) the secretory process of GTs proceeds through discrete morphological and ultrastructural phases; (2) GTs accumulate a distinct set of secondary metabolites, potentially enriched in flavonoid and alkaloid compounds; and (3) transcriptional dynamics reveal conserved and lineage-specific genes involved in formation and metabolite biosynthesis of GTs. Our findings provide a comprehensive multi-omics resource for cucumber GTs research and establish a foundation for future functional studies and biotechnological applications aimed at enhancing trichome-mediated traits in cucumber and related species.

## 2. Results

### 2.1. GT Density on Fruit Varies Significantly Between Cucumber Cultivars

**Figure 1:**
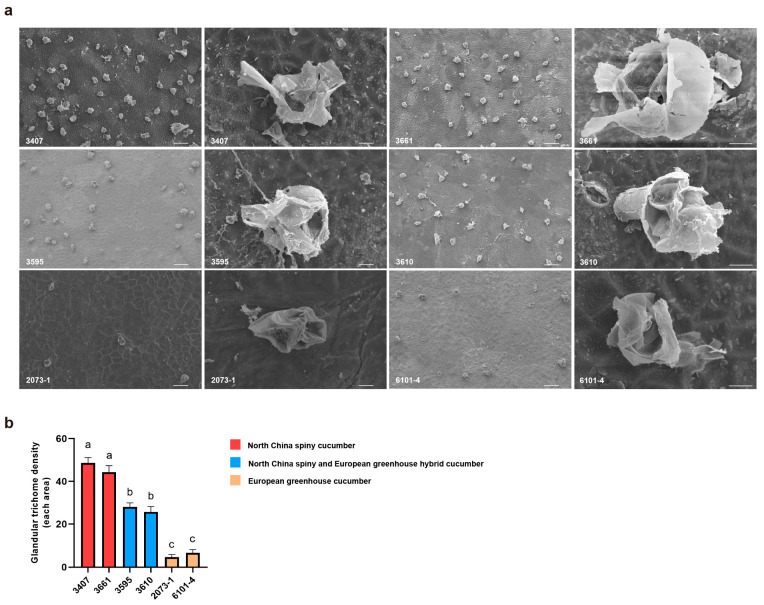
Observation of GTs in different cucumber accessions. (**a**) GT morphology on commercial fruits (10 days post anthesis) across six accessions (3407, 3661: North China spiny; 3595, 3610: hybrid progenies; 2073-1, 6101-4: European greenhouse). Scale bars = 100 μm (1st and 3rd columns); 10 μm (2nd and 4th columns). (**b**) Quantification of GT density in the six accessions. Data are presented as mean values; significant differences letters were determined via one-way ANOVA (*p* < 0.05); *n* = 5.

To explore the diversity of GT traits across different cucumber germplasms, two accessions were selected from each of three groups: North China eco-type cucumber (3407, 3661), European greenhouse eco-type cucumber (2073-1, 6101-4), and their hybrid progenies (3595, 3610). Scanning electron microscopy (SEM) was performed to observe the GTs on commercial fruits (10 days post anthesis). Morphologically, the GTs of all six accessions shared an identical structural composition, consisting of a glandular head and stalk-connected cells; notably, trichome rupture was observed in all accessions at the commercial fruit stage (Figure 1a). We further quantified the GT density of these materials: the results indicated that North China eco-type cucumbers exhibited the highest density, followed by the two hybrid cucumbers, while European greenhouse eco-type cucumbers showed the lowest density (Figure 1a,b). This observation illustrates that distinct germplasm-specific variation exists in GT density among cucumber ecotypes, whereas the fundamental morphological architecture and late-stage developmental progression (e.g., rupture at maturity for exudate release) of these trichomes are highly conserved across genotypes.

### 2.2. The Secretion Process of GTs Can Be Divided into Four Stages

**Figure 2:**
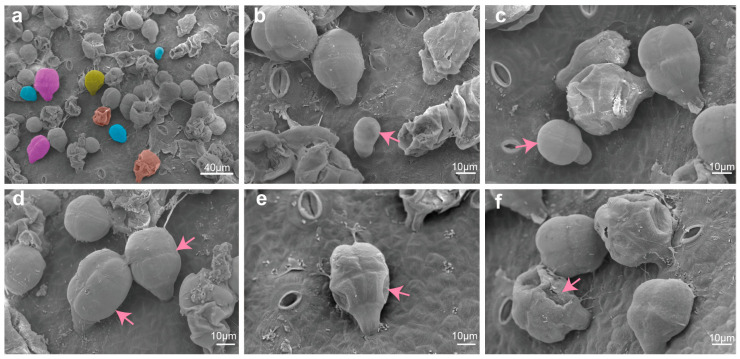
Observation of the secretion process of GTs in North China spiny cucumber. (**a**) GTs on the ovary surface at anthesis; scale bar = 40 μm. (**b**,**c**) Morphogenesis stage; (**d**) active metabolism stage; (**e**) glandular head sunken stage; (**f**) metabolite release stage. Scale bar = 10 μm.

Given that the expansion of peel during cucumber fruit development leads to a significant reduction in GT density, we selected the ovaries of North China eco-type cucumber (3661) at anthesis as samples for SEM observation. GTs with various morphologies were observed (Figure 2a). Cucumber fruit trichomes are naturally asynchronous in their development. Due to differential initiation timing, variable growth rates, and the progression of maturation and senescence. GTs on the same fruit surface can be simultaneously captured in distinct phases. Based on previous descriptions of the developmental process of GTs, we further subdivided the secretion process into four stages. The morphogenesis stage: Trichome precursor cells protrude and expand from the epidermis (Figure 2b), and form glands with four-celled heads through multiple divisions (Figure 2c). The active metabolism stage: During this period, the heads of GTs further enlarge, plump and filled with metabolic substances to be secreted (Figure 2d). The head sunken stage: At this stage, the cells in the heads exhibit an inward depression, while the cuticle remains intact (Figure 2e). The metabolite release stage: With the deepening sinking of the head cells, the cuticle ruptures, facilitating the release of metabolic substances, and leads to severe shrinkage and collapse of the entire glandular head (Figure 2f).

### 2.3. Ultrastructural Changes Occur During the Secretion Process of GTs

**Figure 3:**
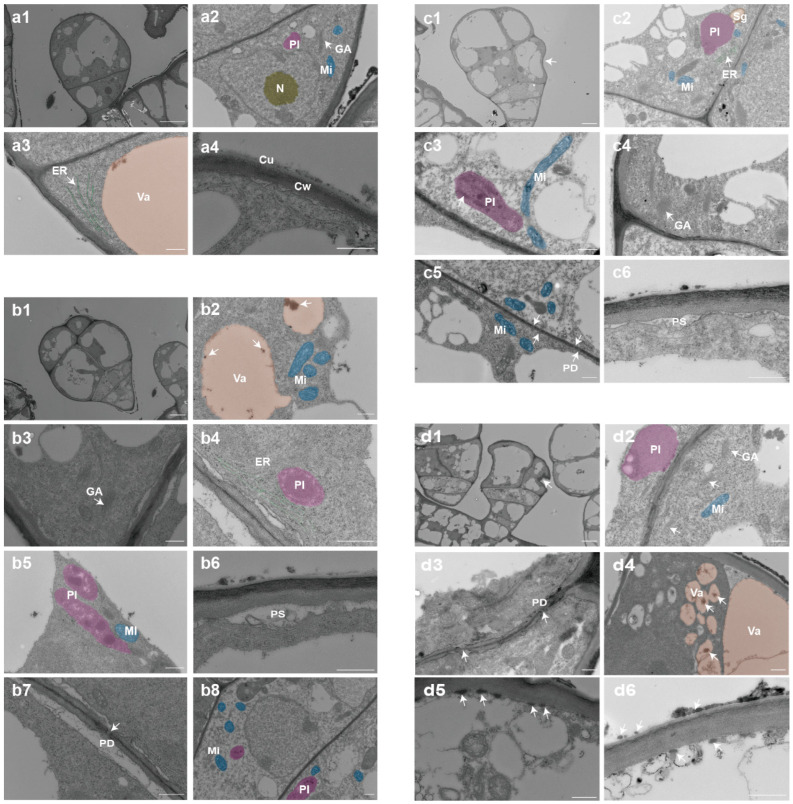
Ultrastructural observation of GTs at different developmental stages in North China eco-type cucumber. (**a1**) GTs before the formation of the four-celled glandular head; scale bar = 5 μm. (**a2**–**a4**) Ultrastructure of GTs at the morphogenesis stage; scale bar = 500 nm. (**b1**) GTs with a four-celled glandular head; scale bar = 5 μm. (**b2**–**b8**) Ultrastructure of GTs at the active metabolism stage; scale bar = 500 nm. (**b2**) Arrow indicates black deposits in vacuoles; (**b3**) arrow indicates vesicles of the Golgi apparatus; (**b7**) arrow indicates plasmodesmata. (**c1**) GTs with a sunken glandular head; scale bar = 5 μm. (**c2**–**c6**) Ultrastructure of GTs at the glandular head sunken stage; scale bar = 500 nm. (**c3**) Arrow indicates lamellar structures within the plastid; (**c5**) arrow indicates plasmodesmata. (**d1**) GTs with a ruptured glandular heads; scale bar = 5 μm. (**d2**–**d6**) Ultrastructure of GTs at the metabolite release stage; scale bar = 500 nm. (**d3**) Arrow indicates cell wall gaps at plasmodesmata; (**d5**,**d6**) arrows indicate metabolite globules accumulated on both sides of the cell wall. Abbreviations: Pl: Plastid; Mi: Mitochondrion; GA: Golgi apparatus; Va: Vacuole; ER: Endoplasmic reticulum; N: Nucleus; Cu: Cuticle; Cw: Cell wall; Sg: Starch granule; PS: Periplasmic space; PD: Plasmodesmata.

GTs possess the ability to synthesize, store, and secrete secondary metabolites, which relies on the support of their internal cellular structures. We observed the ultrastructure of glandular cells at these four stages via TEM. At the morphogenesis stage (Figure 3(a1–a4)), mitochondria, plastids, and underdeveloped Golgi apparatus can be observed (Figure 3(a2)). Sparse endoplasmic reticula are arranged in parallel around the vacuoles, with clear contours and no impurities at the vacuole margins (Figure 3(a3)). Overall, the organelles are small and few. Since the cells are still in the division stage, the cytoplasm is dense with a low degree of vacuolation. A thin cuticle covers the entire surface of the fruit spine, closely adhering to the cell wall, and there is no gap between the cell wall and the plasma membrane (Figure 3(a4)).

At the active metabolism stage (Figure 3(b1–b8)), the degree of cellular vacuolation is high, with organelles increasing in both quantity and volume. The cristae inside mitochondria are visible, and flocculent or clumpy metabolite deposits are observed at the vacuole margins (Figure 3(b2)). The Golgi apparatus consists of multiple stacked flattened cisternae, with small vesicles distributed around them (Figure 3(b3)). The endoplasmic reticulum is abundant and densely studded with ribosomal particles (Figure 3(b4)). Plastids contain plastoglobuli, with reticular structures surrounding them (Figure 3(b5)). The cell wall separates from the plasma membrane, forming a periplasmic space where numerous small vesicles are distributed (Figure 3(b6)). Plasmodesmata can be observed between the glandular head cells (Figure 3(b7)). Mitochondria and plastids are also present in the stalk cells, but they are relatively smaller in size, and the electron density of the cytoplasm is lower (Figure 3(b8)).

At the head sunken stage, small vacuoles gradually fused into large vacuoles (Figure 3(c1)). This fusion led to a high degree of vacuolation, resulting in low cytoplasmic electron density. Some plastids also contained starch grains (Figure 3(c2)). Various organelles were greater in number and larger in volume. The cristae inside mitochondria were clear and showed a high degree of folding (Figure 3(c3)). The Golgi apparatus aggregated near the cytoplasmic membrane (Figure 3(c4)). Plasmodesmata were more clearly visible (Figure 3(c5)), and many small vesicles were present in the periplasmic space (Figure 3(c6)).

At the metabolite release stage (Figure 3(d1)), a large number of organelles such as mitochondria and plastids were still visible. Notably, the number of Golgi apparatuses was greater, and they were often concentrated around the plasma membrane (Figure 3(d2)). The cell wall at the plasmodesmata showed cracks and gaps due to head invagination and metabolite release activities (Figure 3(d3)). The cells exhibited a high degree of vacuolation, with vacuoles generally being large in size. Small vacuoles enclosing flocculent substances also often aggregated and arranged in a reticular pattern, showing a tendency to fuse (Figure 3(d4)). A large number of metabolite globules aggregated near the edge of the cell wall in the periplasmic space, and the metabolites released outside the cells also aggregated in a globular form (Figure 3(d5,d6)).

### 2.4. Preliminary Identification of Metabolite Components in GTs via Histochemical Staining

**Figure 4:**
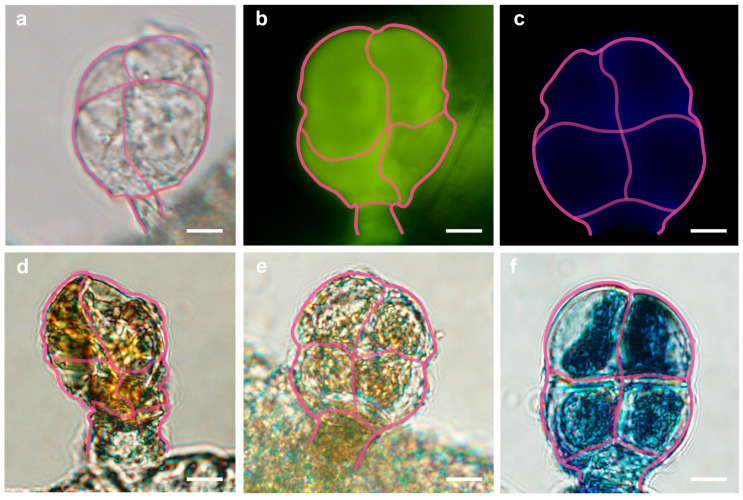
Histochemical staining of GTs of North China eco-type cucumber. (**a**) Unstained GTs under the microscope showing colorlessness. (**b**) GTs exhibiting yellowish–green fluorescence after staining with aluminum chloride-ethanol solution. (**c**) GTs emitting blue fluorescence upon UV induction. (**d**) GTs stained orange with Sudan-III solution. (**e**) GTs stained yellow with iodine–potassium iodide solution. (**f**) GTs stained blue with toluidine blue. Scale bar = 10 μm.

To obtain a preliminary understanding of the major categories of metabolites in GTs, we prepared North China eco-type cucumber ovaries at anthesis and conducted targeted histochemical staining for the metabolites inside GTs. Under an optical microscope, unstained GTs appeared colorless (Figure 4a). GTs stained with aluminum chloride ethanol solution emitted obvious yellow–green fluorescence under ultraviolet induction, indicating the presence of flavonoids (Figure 4b). Under a fluorescence microscope, unstained GTs emitted blue autofluorescence when induced by ultraviolet light, which again indicated the presence of phenols (Figure 4c). Staining with Sudan III solution, the glandular head cells exhibited an orange–red color, indicating that cells are rich in lipids (Figure 4d). Staining with iodine-potassium iodide solution revealed the presence of alkaloids in glandular head cells (Figure 4e). Staining with toluidine blue solution indicated the presence of phenols in the cells (Figure 4f). These results were further validated by metabolomic sequencing.

### 2.5. Identification of 744 Metabolite Components in Six Samples of GTs via LC-MS

To further detect the metabolite components in GTs, we collected ovaries at anthesis from three European greenhouse eco-type cucumber samples (2073-1) and three North China eco-type cucumber samples (3661) to extract GT metabolites. Ultra-high-performance liquid chromatography (UHPLC) was used for chromatographic separation of the GT extracts, and a triple quadrupole mass spectrometer was employed to perform mass spectrometry analysis and data acquisition on the extract samples under multiple reaction monitoring (MRM) mode.

**Figure 5:**
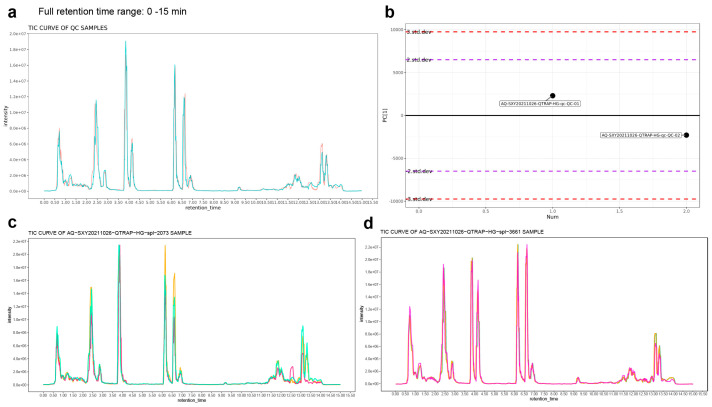
Targeted metabolite detection results of GTs in European Greenhouse eco-type cucumber and North China eco-type cucumber. These TICs represent the full 0–15 min retention time window of the UHPLC-MS method. (**a**) Total ion current chromatograms (TICs) of QC samples. (**b**) PCA-X one-dimensional distribution plot of QC samples. (**c**) Total ion current chromatograms (TICs) of GT samples in European Greenhouse eco-type cucumber (2073), *n* = 3. (**d**) Total ion current chromatograms (TICs) of GT samples in North China eco-type cucumber (3661), *n* = 3.

The retention times and peak areas of the total ion current chromatograms (TICs) of quality control (QC) samples overlapped well, indicating excellent stability of the instrument (Figure 5a). All QC samples were within the range of two standard deviations (SD), demonstrating that the systematic error of the LC-MS detection data was within a controllable range (Figure 5b). Additionally, the TIC profiles of GT samples from European greenhouse eco-type cucumber (Figure 5c) and North China eco-type cucumber (Figure 5d) were highly consistent, which further verified the good reproducibility of the experimental system. Overlapping peaks demonstrate excellent retention time stability and analytical reproducibility. After analyzing and processing the detection data of cucumber GT extract samples using LC-MS data analysis software (SCIEX Analyst Work Station Software Version 1.6.3) and comparing the results in databases (KEGG, HMDB, METLIN, CAS, Pubchem, and chEBL), a total of 744 metabolites were qualitatively identified in six samples of GTs (Supplemental Table S1).

### 2.6. No Significant Difference in Metabolite Composition of GTs Between Test Materials

**Figure 6:**
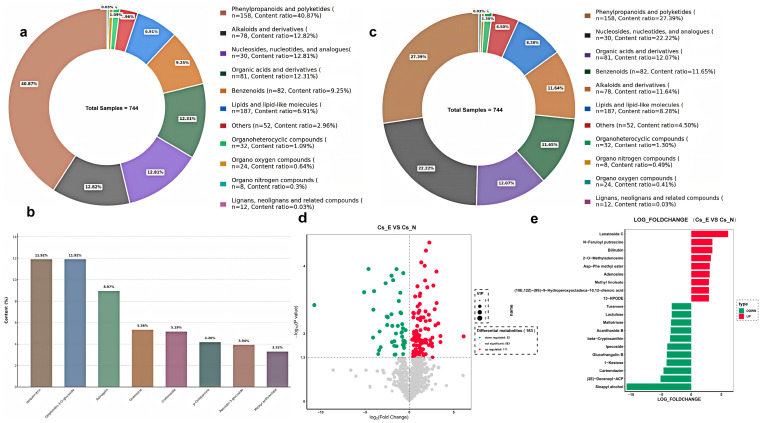
Metabolite profiles analysis of GTs between European greenhouse eco-type and North China eco-type cucumbers. (**a**) Classification and composition of 744 metabolites in GTs of North China eco-type cucumber. (**b**) Content distribution of key metabolites (content > 3%) in GTs of North China eco-type cucumber. (**c**) Classification and composition of 744 metabolites in GTs of European greenhouse eco-type cucumber. (**d**) Fold change analysis of metabolites in GTs between two eco-types. (**e**) Volcano plot of differential metabolites in GTs between two eco-types.

These metabolites in North China eco-type cucumber were classified into 11 major categories based on their chemical structures: phenylpropanoids and polyketides (158, 40.87%); alkaloids and their derivatives (78, 12.82%); nucleosides/nucleotides and their analogs (30, 12.81%); organic acids and their derivatives (81, 12.31%); benzenoids (82, 9.25%); lipids and lipid-like molecules (187, 6.91%); organoheterocyclic compounds (32, 1.09%); organo oxygen compounds (24, 0.64%); organo nitrogen compounds (8, 0.30%); lignans, neolignans and related compounds (12, 0.03%); others (52, 2.96%) (Figure 6a). Further analysis revealed that among the 158 phenylpropanoid and polyketide metabolites, 114 were flavonoids, accounting for 40.39% of the total content (Supplemental Table S1). There were eight metabolites with a relative content of more than 3% (Figure 6b), including four phenylpropanoids and polyketides—Isoquercitrin (11.92%), Delphinidin-3-O-glucoside (11.92%), Astragalin (8.97%) and Paeoniflorin-3-glucoside (3.94%); two alkaloids and their derivatives—Crotonoside (5.19%) and Methyl anthranilate (3.32%); one benzenoid—p-Hydroxyphenylethanolamine (4.20%); and one nucleoside/nucleotide and its analog—Guanosine (5.36%). Although lipids and lipid-like molecules had the largest number of species (up to 187), they had no advantage in relative content. They mainly included 39 fatty acyls (3.41%) and 110 terpenoids (2.86%). Terpenoids are one of the most reported important secondary metabolites in GTs of medicinal plants. However, according to our results, although terpenoids in cucumber GTs were rich in species, their total content only accounted for 2.86%, with Miltirone being the most abundant, accounting for 0.94% of the total content (Supplemental Table S1).

To compare the metabolite profiles of GTs between European greenhouse eco-type cucumbers and North China eco-type cucumbers, we analyzed their metabolite composition. Our results revealed that the categories of metabolites were identical between the two ecotypes, with only subtle quantitative variations in a subset of metabolites (Supplemental Tables S1 and S2), indicating a highly conserved core metabolite class composition across the two germplasms. Notably, phenylpropanoids and polyketides were the most abundant metabolite class in both ecotypes, and their content was higher in North China eco-type cucumbers than in European greenhouse eco-type cucumbers (Figure 6a,c). These findings, together with our earlier observation of conserved GT morphology, suggest that while cucumber germplasms of distinct genetic backgrounds exhibit significant differences in GT abundance, the fundamental metabolic functions of these trichomes are evolutionarily conserved.

Among these 744 metabolites, 581 metabolites showed no significant difference in content between the two types cucumbers, while 163 exhibited significant variations. Specifically, 52 metabolites were downregulated in European greenhouse eco-type cucumber, while 111 metabolites were upregulated in this cultivar compared to North China eco-type cucumbers (Figure 6d). The fold changes in the top 20 differential metabolites (ranked by relative content difference) were visualized in the form of a bar chart. The ordinate represents the names of differential metabolites, and the abscissa represents the logarithmic values of the fold changes in differential metabolites. Red indicates upregulation in European greenhouse eco-type cucumbers, while green indicates downregulation (Figure 6e). The most significantly upregulated substance in European greenhouse eco-type cucumbers was Lanatoside C. Lanatoside C is a steroid substance and a fast-acting cardiac glycoside, which can be used to treat congestive heart failure and arrhythmia [21]. The most significantly downregulated metabolite was sinapyl alcohol—a critical lignin biosynthesis monomer. Lignin plays an essential role in plant cell wall assembly and the maintenance of intercellular structure [22]. Given that the GT density of European greenhouse eco-type cucumber is substantially lower than that of North China eco-type cucumber, their demand for lignin (required for trichome cell wall construction) is correspondingly reduced. This is ultimately reflected in the marked downregulation of sinapyl alcohol, a lignin monomer, at the metabolic level. These metabolomic data provide direct biochemical evidence for the phenotypic difference in GT density between the two eco-types.

### 2.7. Identification of GT-Associated Genes via Multi-Transcriptome Analysis and Functional Validation in Cucumber

**Figure 7:**
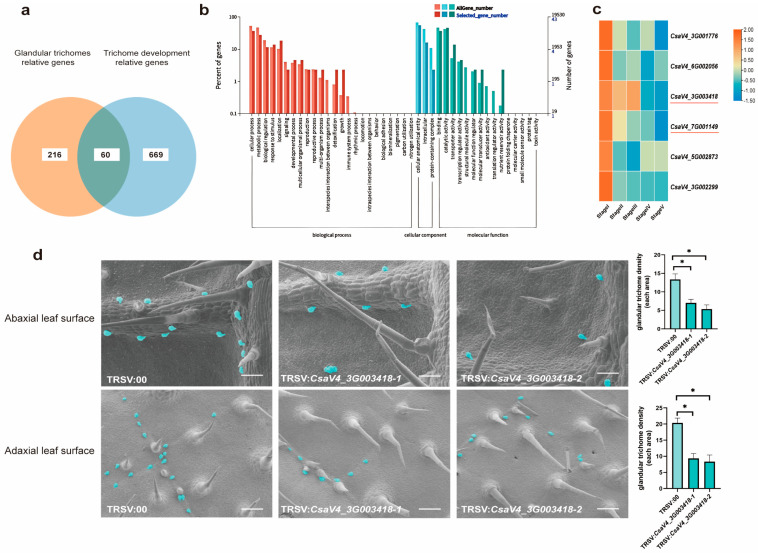
Identification and functional validation of genes regulating cucumber GT formation. (**a**) Venn diagram showing the overlap between GT-relative genes and trichome development genes. (**b**) Gene Ontology (GO) enrichment analysis of the 60 candidate GT-relative genes. (**c**) Heatmap of expression patterns for 6 candidate genes. The genes marked with red lines represent the potential candidate genes we screened for regulating glandular trichome development, and they exhibit consistent expression patterns. (**d**) GT phenotype of VIGS plants: TRSV:00, TRSV: *CsaV4_3G003418-1*, and *CsaV4_3G003418-2*. The bar plots below quantify GT density (Student’s *t*-test, * *p* < 0.05, *n* = 5). Scale bar = 100 μm (top row) and 50 μm (bottom row).

By integrating multi-omics datasets with our laboratory’s prior work on cucumber trichome development [4,23], we identified 60 candidate genes implicated in GT formation and metabolism (Figure 7a, Supplemental Table S3, Appendix A). To characterize the functional landscape of these genes (putatively regulating secondary metabolite biosynthesis and secretion in GTs), we performed Gene Ontology (GO) enrichment analysis. Within the biological process category, these candidates were significantly enriched in metabolic process (Figure 7b), highlighting their core role in secondary metabolite biosynthesis. In the molecular function category, catalytic activity and binding were the most highly enriched terms (Figure 7b). This functional bias suggests that most of these genes encode enzyme-like proteins, which directly mediate biosynthetic reactions of specialized metabolites in this tissue. We found that *CsMIF3* (*CsaV4_1G000245*, a homolog of *Arabidopsis MINI ZINC FINGER 3* and a member of the ZF-HD TF family) was present in the 60 candidate genes, and this gene has been previously reported to mediate the invagination of the GT heads [23]. This result confirms that the identified candidate gene set is indeed involved in the development of GTs, and further validates our research strategy.

To narrow down genes involved in early GT development, we further analyzed their expression patterns and screened six genes with high expression at the cucumber trichome initiation stage (Figure 7c). This expression profile implies their potential roles in driving GT formation. Prior studies in our lab validated that VIGS-mediated silencing of *CsaV4_7G001149* (*Arabidopsis* homolog of *membrane bound O-acyl transferase1/MBOAT1*) reduced its expression and caused a significant decrease in GT density [23]. We next selected *CsaV4_3G003418* (*Arabidopsis* homolog of putative aldehyde reductase) for functional validation, as it shares a nearly identical expression pattern with *CsaV4_7G001149* (Supplemental Figure S1a, Supplemental Table S4). VIGS assays showed that silencing *CsaV4_3G003418* led to a marked reduction in GT density on both abaxial and adaxial leaf surfaces (Figure 7d, Supplemental Figure S1b). These results confirm that *CsaV4_3G003418* also controls GT density in cucumber.

## 3. Discussion

### 3.1. The Secretory Process of GTs in Cucumber

By examining the morphological features of GTs via SEM, we delineated their secretion process as four distinct stages: morphogenesis, active metabolism, head sunken, and metabolite release. The first two stages primarily encompass the structural development of glands and the accumulation of metabolites, whereas the latter two stages mediate metabolite release. Notably, metabolite excretion ultimately induces severe shrinkage and collapse of the entire glandular structure. Drawing on previous investigations of GTs across diverse plant species, we summarized three canonical modes of secondary metabolite secretion. (A) Cuticular rupture: For example, in mature GTs of Doronicum, metabolites accumulate to a threshold level in the subcuticular space between the cell wall and cuticle, prior to being released through cuticular rupture [24]. (B) Micropore-mediated release: As observed in Robinia pseudoacacia, the cuticle at the apex of GTs during the secretory phase contains minute pores, which facilitate the release of metabolites sequestered in the subcuticular space [25]. (C) Contact-triggered release: GTs of Cucurbita moschata, for instance, release metabolites upon contact with setae, epidermal cells, or other trichomes [26]. However, the secretion mode of secondary metabolites by cucumber GTs deviated from these three types. We propose that the secretion of GTs constitutes an autonomous process.

During the active secretion stage, mitochondria and other organelles in GTs proliferate, with active vesicle trafficking and enhanced plasmodesmata. Metabolites are transported to the periplasmic space and released extracellularly. As secretion proceeds, reduced cytoplasmic density, increased vacuolation, and plasma membrane separation occur, causing autonomous collapse of the gland head. This process represents an autonomous, programmed secretion driven by cellular metabolism, substance transport, and structural changes, constituting a novel secretory model for GTs.

As the most abundant organelles in GTs, mitochondria mainly function to provide energy for intracellular metabolic activities and substance transport. Therefore, with the progression of the secretion process, mitochondria showed an increase in number and volume, and their cristae became more well-developed. It has been reported that secretory cells containing chloroplasts (e.g., long-stalked GTs of tobacco) can perform photosynthesis, providing precursors and energy to produce secretory substances through the degradation of carbohydrates. However, no chloroplasts were found in GTs of cucumber, which is consistent with the colorless appearance of fresh, unstained GTs observed via hand-sectioning. This may also imply that mitochondria need to assume more energy supply tasks. Plastids were also abundant during the secretion process of GTs. With the progression of the trichome secretion process, plastids in the cells increased significantly, exhibiting a high degree of structural complexity and often forming crystalloid-like structures internally. In its cells, abundant endoplasmic reticulum was often distributed around plastids, vacuoles, and the plasma membrane. The abundance of ER is not only related to the transport of metabolic substances from plastids, vacuoles, and other sites to the plasma membrane but also associated with the synthesis and processing of metabolic substances. Molecular biological studies have shown that multi-enzyme complexes or metabolites are present on the ER membrane, catalyzing the sequential reactions of flavonoid and phenylpropanoid metabolism [27,28]. In addition, rough endoplasmic reticulum (RER) is also involved in the production of phenolic compounds [29]. In the GTs of *Millingtonia hortensis*, Golgi vesicles with smooth membranes secrete polysaccharides into the periplasmic space, which then accumulate in the cell wall [30]. However, our LC-MS analysis results revealed that the polysaccharide content in the metabolites of cucumber GTs is extremely low. Therefore, the specific relationship between the presence of these organelles and the production and transport of metabolites in cucumber GTs requires further in-depth investigation.

Our study is the first to divide the secretory stages of GTs and observe the changes in organelles at each stage via TEM. And we systematically and comprehensively described the changes during the secretory stages, which provides a theoretical foundation for the research on GTs.

### 3.2. Metabolite Composition of Cucumber GTs

Histochemical staining facilitated the localization of the main chemical classes of metabolites in GTs. The results indicated that GTs contain flavonoids, phenols, alkaloids, and lipids. Further LC-MS analysis confirmed the findings, and leading to the qualitative identification of a total of 744 metabolites, which were classified into 11 major categories. Among these, phenylpropanoids and polyketides accounted for the highest relative content, while lipids and lipid-like substances exhibited the greatest diversity in terms of species.

Flavonoids are widely distributed secondary metabolites that play crucial roles in helping plants resist environmental stresses. Additionally, flavonoids are indispensable for human health due to their extensive pharmacological activities and are used as raw materials in the production of food additives. Astragalin, in particular, acts as an antioxidant in organisms, effectively scavenging reactive oxygen species (ROS) and exerting anti-inflammatory, bactericidal, anti-cancer (inhibiting cancer cell proliferation and invasion), and therapeutic effects on chronic heart failure, among others [31]. Alkaloids possess strong biological and pharmacological activities; 78 alkaloids and their derivatives were detected in the metabolites of GTs, with crotonoside having the highest relative content. In research on plant GT metabolites, terpenoids have long attracted significant attention and are regarded as one of the most widely distributed and diverse specialized secondary metabolites in medicinal plants. However, terpenoid content was not abundant in the detection results of this study. This may be attributed to the most terpenoids are volatile compounds. Volatilization loss may occur during the collection of GTs using the bead-beating method.

In our results, the metabolite components are highly conserved in different ecotypes, with only differences in content. The conservation of metabolite components in GTs has very important biological significance. Conserved metabolite components are generally core defensive substances retained during the long-term evolution of plants, which are crucial for plant survival and can resistance against many stresses. On the other hand, the conservation of metabolic profiles reflects the functional stability of GTs as defensive organs, ensuring defensive efficacy under diverse conditions.

### 3.3. Biotechnological Implications and Future Perspectives

This study comprehensively analyzed the secretory stages, ultrastructural dynamics, and metabolite composition of GTs in cucumber, providing a foundational resource for diverse biotechnological applications. First, the identification of key metabolites with well-characterized pharmacological and agrochemical activities—such as astragalin, crotonoside, and lanatoside C—highlights the potential of cucumber GTs as a sustainable source of high-value natural products. These compounds could be harnessed for the development of nutraceuticals, plant-based therapeutics, or natural preservatives. Second, the method established herein for GT isolation and metabolite extraction can be further optimized and scaled up for industrial bioreactor-based production, leveraging GTs as miniaturized natural bioreactors. Third, SEM observations and metabolomic sequencing results revealed that despite significant variations in GT density among different accessions, their structure and the metabolite composition are highly conserved. Additionally, the finding that *CsaV4_3G003418* regulates GT abundance provides insights for developing GT clusters as “plant factories”. In the future, we can use transgenic technology to increase gene expression levels, thereby obtaining more metabolites, including flavonoids and terpenoids. These changes will strengthen plant resistance to diseases, insects and environmental stresses, and reduce the application of pesticides. Meanwhile, elevated metabolite levels can improve the nutritional quality and antioxidant activity of cucumber fruits, and enhance commercial value.

Collectively, the results of this study lay a solid foundation for future broader and deeper investigations into the functions of cucumber GTs, as well as the molecular regulatory mechanisms and metabolic pathways underlying their metabolites. Moreover, the identification of key genes controlling the quantity of these “metabolic factories” will accelerate the translation of these findings into tangible bioproduction outcomes.

## 4. Materials and Methods

### 4.1. Experimental Materials and Planting Methods

In this study, we observed the GTs of six cucumber accessions: 3407, 3661, 3595, 3610, 2073-1, and 6101-4. Ultimately, we selected the North China eco-type cucumber (3661) and the European greenhouse eco-type cucumber (2073-1) for metabolome sequencing. All accessions used were preserved in our laboratory.

Plump cucumber seeds were immersed in warm 50–55 °C water, stirred continuously for 3–4 h. After removal, seeds were wrapped in moist gauze and germinated in the dark at 28 °C for 24 h. Seeds with uniform germination were sown in a mixed substrate of peat moss and vermiculite (1:1, *v*/*v*), and cultured under conditions of 26 °C (day)/18 °C (night) with a 16 h photoperiod (light intensity of 200 μmol·m^−2^·s^−1^). When the seedlings grew to the two-leaf and one-heart stage (about one week after sowing), healthy seedlings were selected and transplanted to a greenhouse at the Dongbeiwang Agricultural Base in Beijing. Subsequent field management was performed, including regular irrigation and fertilization. When the plants reached approximately 1.5 m in height, their ovaries at anthesis were used for subsequent experiments (about 2 months after transplanting).

### 4.2. Scanning Electron Microscope (SEM) Observation

Fresh pericarp sections (5 × 5 × 1–2 mm) from ovaries at anthesis of approximately two-month-old plants were fixed in 3% glutaraldehyde (in 0.1 M PBS, pH 6.8) at 4 °C for 24 h. After rinsing three times for 15 min with PBS, samples were post-fixed with 1% osmium tetroxide, dehydrated through a graded ethanol series (50–100%), and transferred to acetone followed by isoamyl acetate. Samples were critical-point dried (CO_2_), sputter-coated with 10 nm gold, and examined with a Hitachi S-4700 SEM, Hitachi High-Tech Corporation, Tokyo, Japan at 2 kV.

### 4.3. Transmission Electron Microscopy (TEM) Observation

Fresh pericarp sections (4 × 4 × 1–2 mm) from ovaries at anthesis of approximately two-month-old plants were fixed in a mixture of 4% glutaraldehyde and 3% paraformaldehyde (0.1 M PBS, pH 7.2) at 4 °C for 24 h. After washing with PBS, specimens were post-fixed in 1% osmium tetroxide, dehydrated in a graded ethanol series, and embedded in epoxy resin via propylene oxide. Ultrathin sections were stained with uranyl acetate and lead citrate, then observed using a transmission electron microscope (ECNAI G2 12 TEM, FEI Company, Brno, Czech Republic).

### 4.4. Tissue Chemical Staining

Take healthy cucumber ovaries at anthesis of approximately two-month-old plants for free-hand sectioning. After staining with histochemical reagents, observe the staining of GTs under a microscope. Directly observe and photograph fresh, unstained GTs using an OLYMPUS IX71 Inverted Microscope, Olympus Corporation, Tokyo, Japan. Detect lipids using Sudan III staining solution. Detect alkaloids using iodine-potassium iodide solution. Detect phenols using toluidine blue solution. After inducing fresh, unstained GTs with ultraviolet light, observe the autofluorescence of phenolic substances under a Nikon Ti-E inverted fluorescence microscope, Nikon Instruments Inc., Tokyo, Japan. Detect flavonoids using aluminum chloride ethanol solution; after ultraviolet light induction, observe and photograph under a Nikon Ti-E inverted fluorescence microscope.

### 4.5. GT Separation and Enrichment

A pre-cooled buffer (300 mL) containing 200 mM of sorbitol, 50 mM of Tris-Cl, 20 mM of sucrose, 10 mM of KCl, 5 mM of MgCl_2_, 5 mM of succinic acid, 1 mM of EGTA, 0.5 mM of Na_2_HPO_4_, and 0.015% of Triton X-100 was prepared. Thirty freshly harvested cucumber ovaries at anthesis of approximately two-month-old plants were cut into 1 cm segments, placed in a bottle with 150 mL of cold buffer and 30 g of 1 mm glass beads, and shaken vigorously for about 5 min until the pericarp turned dark green and numerous non-glandular trichomes fell off. The mixture was quickly poured over a steel sieve, rinsed with buffer, and sequentially filtered through 110-, 250-, and 320-mesh sieves. The filtrate below the 320-mesh sieve was collected, transferred to a 50 mL tube, and centrifuged at 10,000× *g* for 5 min. The pellet, containing enriched GTs, was resuspended in 1 mL buffer, transferred to a 2 mL tube, vortexed for 30 s, and quantified with a hemocytometer. After confirming a yield of no less than 10^6^ spines, the suspension was centrifuged again (10,000× *g*, 5 min), the supernatant was discarded, and the pellet was snap-frozen in liquid nitrogen and stored at −80 °C [23].

### 4.6. Extraction of Metabolites from GTs

Thaw the stored GT samples, then centrifuge them at 12,000 rpm and 4 °C for 15 min and remove the supernatant. Take out the precipitate, mix it evenly, weigh 50 mg of it and place it in a 1.5 mL centrifuge tube. Add 700 μL of pre-cooled extract (−40 °C), where the extract is a mixture of methanol and water at a volume ratio of 3:1 and contains the internal standard 2-chlorophenylalanine. Vortex for 30 s, then homogenize at 35 Hz for 4 min and perform ultrasonic treatment in an ice-water bath for 5 min. Repeat the homogenization and ultrasonic process three times in total, then place the tube on a mixer and incubate overnight at 4 °C. Centrifuge the sample at 12,000 rpm and 4 °C for 15 min. Carefully collect the supernatant and filter it through a 0.22 μm microporous membrane. Dilute the supernatant 10-fold with the extract; vortex for 30 s. Take 80 μL from each sample, mix them to form a Quality Control (QC) sample, and store it in a −80 °C refrigerator until instrumental analysis.

### 4.7. Detection of Metabolite Components in GTs

Chromatographic conditions: An EXION LC System (SCIEX) ultra-high performance liquid chromatograph (AB Sciex LLC, Framingham, MA, USA) was used, and the target compounds were chromatographically separated through a Waters UPLC column, Waters Corporation, Milford, MA, USA (diameter × length: 2.1 × 100 mm, packing material diameter: 1.8 μm). The mobile phase conditions are shown in Table 1.

Mass spectrometric conditions: A SCIEX 6500 QTRAP+ triple quadrupole mass spectrometer (AB Sciex LLC, Framingham, MA, USA) equipped with an IonDrive Turbo V ESI ion source (AB Sciex LLC, Framingham, MA, USA) was used, and mass spectrometric analysis was performed in multiple reaction monitoring (MRM) mode (All experiments described above were performed by Ovison Gene Technology Co., Ltd., Beijing, China). The ion source parameters are as follows: positive/negative ion spray voltage (ISVF) +5500/−4500 V, curtain gas (CUR) 35 psi, ion source heating temperature 400 °C, ion source gas 1 (Gas1) 60 psi, ion source gas 2 (Gas2) 60 psi, and decluttering potential (DP) ± 100 V. In the triple quadrupole, each ion pair was scanned and detected according to the optimized decluttering potential and collision energy.

The screening criteria for differential metabolites were set as follows: a *p*-value < 0.05 from Student’s *t*-test, combined with a Variable Importance in the Projection (VIP) score ≥ 1.

### 4.8. VIGS Assay and Phenotypic Observation

To investigate the functions of candidate genes, we employed a modified TRSV-based VIGS system. The detailed procedure was as follows: A 300–500 bp fragment of the target gene’s coding sequence (CDS) was cloned into the SnaBI restriction site of the pTRSV2 vector, and the resulting recombinant construct was subsequently introduced into GV3101. Use *CsPDS* as the positive control. After cucumber seeds had germinated for 36–48 h, they were immersed in a mixed *Agrobacterium* suspension (containing both pTRSV1 and pTRSV2 with distinct target fragments, OD_600_ = 0.8–1.0) and subjected to vacuum infiltration at −900 kPa for 10 min. The treated seeds were then transferred to MS solid medium supplemented with 100 μM of acetosyringone, where they were cultured for 4–5 days. Seedlings were transplanted into soil and grown for 15 d, after which phenotypic characterization was performed. Tissue samples were collected from the same position on the first true leaf of each seedling; these samples were used for phenotypic observation via SEM and gene expression quantification using RT-qPCR. The GT density of each silenced line was quantified based on five different fields of view [32].

### 4.9. RT-qPCR

Total RNA was extracted using an RNA extraction kit (Huayueyang, Beijing, China). cDNA synthesis was performed with a PrimeScript Reagent Kit containing gDNA Eraser (TaKaRa, Shiga, Japan) for reverse transcription. RT-qPCR was conducted on a 96-well plate using an ABI 7500 Real-Time PCR System (Applied Biosystems, Waltham, MA, USA) with SYBR Premix Ex Taq (TaKaRa, Shiga, Japan). Each sample included 3 biological replicates and 3 technical replicates. *CsUBI* was used as the reference gene.

## 5. Conclusions

Division of secretion stages: We systematically divided the GT secretion process into morphogenesis, active metabolism, head depression, and metabolite release.

Novel secretion mechanism: Based on ultrastructural observations, we proposed a unique autonomous secretion model for cucumber GTs—a process distinct from canonical cuticular rupture or pore-based release mechanisms reported in other plants.

Comprehensive metabolite atlas: We identified 744 GT metabolites (11 major categories), revealing a rich and functionally diverse chemical repertoire dominated by phenylpropanoids and flavonoids, with potential applications in nutrition, pest management, and natural product development.

Gene-metabolite integration: We screened 60 candidate genes linked to GT formation and metabolism (e.g., *CsaV4_3G003418* regulating abundance), providing a genetic toolkit for future engineering of trichome-mediated traits in crops.

## Figures and Tables

**Table 1:** Liquid chromatography mobile phase conditions.

Time/min	Flow Rate/µL·min^−1^	A% Water	B% Acetonitrile
0.0	400	98	2
0.5	400	98	2
10.0	400	50	50
11.0	400	5	95
13.0	400	5	95
13.1	400	98	2
15	400	98	2

## Data Availability

The original contributions presented in this study are included in the article/Supplementary Material. Further inquiries can be directed to the corresponding authors.

## Supplementary Materials

The following supporting information can be downloaded at https://www.mdpi.com/article/10.3390/ijms27073276/s1.

## Appendix A

The 60 candidate genes obtained in this study were identified based on previous research results from our laboratory. Previous studies have mined potential genes involved in the development of multicellular trichomes in cucumber using a cotyledon system. Furthermore, potential regulatory genes related to glandular trichomes were identified by isolating glandular trichomes from North China dense-spine type cucumber materials and performing transcriptome sequencing. We obtained 60 regulatory genes involved in the development and metabolism of cucumber glandular trichomes by taking the intersection of these two gene sets (Figure 7a).
