# Supplementary material for: Foundation for Bioproduction: Secretory Stages, Metabolite Profiles and Gene Function of Glandular Trichomes in Cucumber
Source: Int J Mol Sci. 2026 Apr 4;27(7):3276. doi: 10.3390/ijms27073276 (PMC13072923; doi:10.3390/ijms27073276)
Supplement: Supplementary file 1 [file ijms-27-03276-s001.zip › Figure S1. Gene expression model and VIGS-mediated silencing efficiency of target gene.pdf]

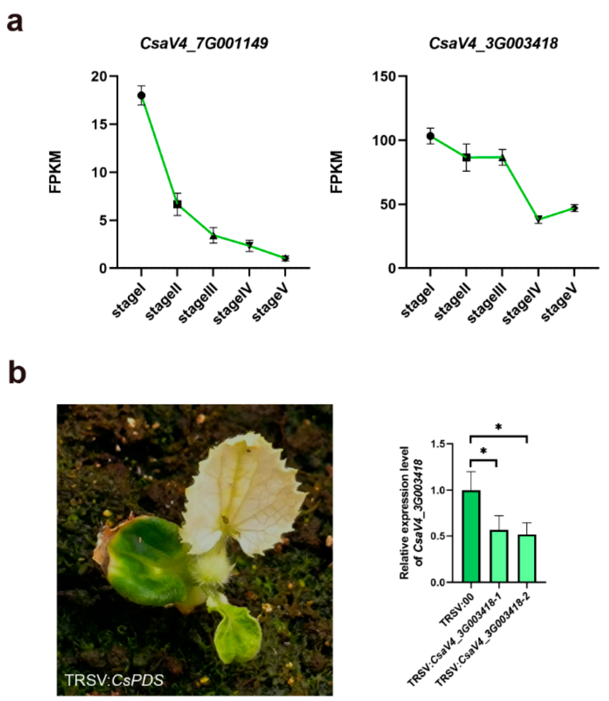

Figure S1. Gene expression model and VIGS-mediated silencing efficiency of target gene. **(a)** FPKM of *CsaV4_7G001149* and *CsaV4_3G003418* (n = 3). **(b)** Phenotype of TRSV:*CsPDS* plant and relative expression level of TRSV:*CsaV4_3G003418* infected plants vs. TRSV:00 plants, confirming successful VIGS. (n = 3; P< 0.05, Student's t-test).
